# Predictors of tumor progression of low-grade glioma in adult patients within 5 years follow-up after surgery

**DOI:** 10.3389/fsurg.2022.937556

**Published:** 2022-10-05

**Authors:** Zhiqiang Yan, Jiang Wang, Qiufeng Dong, Lian Zhu, Wei Lin, Xiaofan Jiang

**Affiliations:** Department of Neurosurgery, Xijing Hospital, Fourth Military Medical University, Xi’an, China

**Keywords:** tumor progression, low-grade glioma, predictors, adult, surgery

## Abstract

**Background:**

Glioma originates from glial cells in the brain and is the most common primary intracranial tumor. This study intends to use a retrospective analysis to explore the factors that can predict tumor progression in adult low-grade gliomas, namely WHO II grade patients, within 5 years after surgery.

**Methods:**

Patients with WHO grade II glioma who were surgically treated in our hospital from February 2011 to May 2017 were included. According to the inclusion and exclusion criteria, 252 patients were included in the final analysis. According to the results of the 5-year follow-up (including survival and imaging review results), patients were divided into progression-free group and progression group. Univariate and multivariate analysis were conducted to investigate the related factors of tumor progression during the 5-year follow-up.

**Results:**

The results of the 5-year follow-up showed that 111 (44.0%) cases had no progress (progression free group, PFG), 141 (56.0%) cases had progress (progression group, PG), of which 43 (30.5%) cases were operated again, 37 cases (26.2%) received non-surgical treatments. There were 26 (10.3%) all-cause deaths, and 21 (8.3%) tumor-related deaths. Univariate and multivariate analysis showed that age >45 years old (OR = 1.35, 95% CI, 1.07–3.19, *P* = 0.027), partial tumor resection (OR = 1.66, 95% CI, 1.15–3.64, *P* = 0.031), tumor diameter >3 cm (OR = 1.52, 95% CI, 1.14–4.06, *P* = 0.017) and no radiotherapy (OR = 1.37, 95% CI, 1.12–2.44, *P* = 0.039) were independent predictors of the progression of tumor during the 5-year follow-up period.

**Conclusion:**

Age >45 years old, partial tumor resection, tumor diameter >3 cm, no radiotherapy are predictors for tumor progression for glioma patients after surgery.

## Introduction

Glioma originates from glial cells in the brain and is the most common primary intracranial tumor. There are four grades in WHO classification, which is I–IV, and the grades I and II are further classified as low grades, and grades III and IV as high grades (1, 2). Grade I gliomas are at very early stage which are often difficult to be detected in clinical practice. According to previous investigation, more than 14,000 glioma patients are diagnosed in the United States each year (3). As for China, the annual incidence of all-grades glioma and malignant glioma is about 3–6.4/100,000, and 5.8/100,000, respectively (4). In the past two decades, due to the progress of diagnostic technique, the number of patients with glioma increased, especially in middle- and old age population (5, 6). Previous study showed that the median age of patients with glioblastoma was 64 years when diagnosed, and that of anaplastic glioma was 45 years. The 5-year mortality rate of glioma patients is very high, and rank the third among all malignant tumor after pancreatic cancer and lung cancer (7). The pathogenesis of glioma remains unclear, and the only confirmed risk factor is exposure to high-dose ionizing radiation (8). Low-grade gliomas mainly astrocyte origin or oligodendrocyte origin. It often occurs on the regular health screen and accounts for approximately 30% of all gliomas (9). Compared with high-grade gliomas, low-grade gliomas are more likely to occur in young people who are at aged 30–40 years old (10). Low-grade glioma has good differentiation, slow growth, and low invasiveness, resulting in a better prognosis with longer survival with a median overall survival (OS) of 7 years (average survival 5–14 years) (11). The main treatment for low-grade gliomas is surgery with total or partial resection. Unfortunately, there are still many patients who relapse or continue to progress after surgery, leading to poor outcomes. Early identification of high-risk patients with state-of-the-art strategies has important clinical significance (12). This study intends to use retrospective analysis to explore related risk factors that can predict tumor progression in grade II glioma patients within 5 years after surgery.

## Materials and methods

### Study population

This study mainly observed the 5-year follow-up results and influencing factors of patients with WHO grade II glioma. We retrospectively analyzed patients who underwent surgery for glioma for the first time in our hospital from Jun 2007 to May 2017. Inclusion criteria: (1) Age over 18 years; (2) A clear diagnosis of primary glioma with WHO grade II; (3) First time surgery for glioma. Exclusion criteria: (1) Combined with other cancers or other benign brain tumor; (2) Combined with severe heart, renal, or liver failure, or multiple organ dysfunction syndrome; (3) Combined with rheumatic disease; (4) Long-term use of glucocorticoids for other diseases immediate before or after surgery; (5) Long-term use of immunosuppressive agents for other diseases 3 months before or after surgery. According to the inclusion and exclusion criteria, a total of 252 patients were included in the final analysis. All procedures involving human participants in this study are in compliance with the Declaration of Helsinki (revised in 2013). This study was approved (Approval number: KY20222151) by the Ethics Committee of Xijing Hospital, Fourth Military Medical University and the informed consent of all patients was obtained.

### Surgery and adjuvant therapy

In this study, all patients underwent surgical resection by the same surgery team. Among the included patients, 137 (54.4%) underwent extended tumor resection or total resection, 115 patients (45.6%) received partial resection. To improve the efficacy of treatment, 43 patients (17.1%) received radiotherapy, 38 patients (15.1%) received chemotherapy, 81 patients (32.1%) received both radiotherapy and chemotherapy, and 90 (35.7%) patients did not receive both radiotherapy and chemotherapy.

### Follow-up

All patients underwent regular follow-up at 1 month, 3 months, 6 months, and 12 months after surgical operation, and followed up annually thereafter. During the follow-up, clinic visits were required when the patient's condition changed. Follow-up items included: repeated brain MRI examination, repeated EEG examination, laboratory test included blood cell count, hemoglobin concentration, alanine aminotransferase, aspartate aminotransferase, total bilirubin, direct bilirubin, blood urea nitrogen, serum creatinine, serum uric acid, and electrolyte level. Primary endpoints included all-cause death and tumor-related death and secondary endpoints include syncope, seizure, and cerebral hemorrhage.

### Data collection

After the patients were included finally, the clinical data before surgery were collected. Demographic data included age, gender. Medical history information included diseases, smoking, alcohol use, weight, height, blood pressure, heart rate, and laboratory test results, MRI imaging information (tumor size and location). Tumor progression was defined as: recurring tumor signals at the previous surgical site, the remaining tumor tissue at the first time surgery was larger than the results of repeated MRI examination, and postoperative imaging-confirmed complications related to surgery and deaths.

### Statistical analysis

SPSS 23.0 statistical software (USA, IBM) was used for statistical processing. Quantitative data are tested for normal homogeneity before analysis. Data that conforms to the normal distribution are represented as mean ± standard deviation (SD), and the comparison between groups is conducted by the student *t*-test; those do not conform to the normal distribution are represented as median and the comparison between groups is conducted by the rank sum test. Qualitative data are represented as number and percentages, and comparisons between groups are conducted by *Χ*^2^ test or Fisher's exact test. Univariate and multivariate analysis were used to analyze factors associated with the prognosis of patients with low-grade glioma. A two-sided *P* < 0.05 means the difference is considered statistically significant.

## Results

### Five-year follow-up outcomes

All patients received at least 5 years of follow-up. Within five years after surgery for glioma, 107 (44.0%) cases did not progress (progression free group, PFG), 145 (56.0%) cases progressed (progression group, PG), of which 43 (30.5%) cases received surgery again, 37 (26.2%) received non-surgical treatment. There were 26 cases (10.3%) of all-cause deaths, and 21 (8.3%) tumor-related deaths. There were another 11 patients (4.4%) had symptom aggravation but not receive further MRI examination (Table 1).

**Table 1:** Outcome within 5-year follow-up (*n* = 252).

Adverse events	Number	Percentage
Progression (*n*, %)	141	56.0
Repeated surgery (*n*, %)	43	30.5
Non-surgery treatment (*n*, %)	37	26.2
Tumor-related death (*n*, %)	21	8.3
All-cause death (*n*, %)	26	10.3
Symptom aggravation (*n*, %)	11	4.4

### Baseline characteristics comparison between PFG and PG

Among the 252 patients, 134 (53.2%) were male and 118 (46.8%) were female. The age was 18–73 years, with an average of 41.6 ± 10.8 years old. Among them, 151 cases (59.9%) were under 45 years old, 88 cases (34.9%) were 46–65 years old, and 13 cases (5.1%) were over 65 years old. The baseline data comparison between PFG and PG is shown in Tables 2, 3. The results revealed that statistical difference existed between PFG and PG regarding the following characteristics: age, the proportion of smoking and drinking patients, the neutrophil/lymphocyte ratio, tumor size, and surgery range (*P* < 0.05).

**Table 2:** Baseline characteristics comparison between PFG and PG.

	PG (*n* = 145)	PFG (*n* = 107)	*t*/*X*^2^ value	*P*-value
Age (years)	43.2 ± 11.5	37.3 ± 9.9	4.267	<0.01
≥45	67 (46.2)	34 (31.8)	5.339	0.021
<45	78 (53.8)	73 (68.2)		
Male (*n*, %)	73 (50.3)	61 (57.0)	1.098	0.295
BMI (kg/m^2^)	24.1 ± 3.6	23.5 ± 3.9	1.262	0.208
Smoking (*n*, %)	55 (37.9)	26 (24.3)	5.246	0.022
Alcohol (*n*, %)	61 (42.0)	30 (28.0)	5.254	0.022
WBC (×10^9^/L)	7.3 ± 2.6	6.9 ± 2.9	1.149	0.252
NEU (×10^9^/L)	4.4 ± 1.9	4.1 ± 2.0	1.211	0.227
LYM (×10^9^/L)	2.3 ± 0.8	2.4 ± 0.6	1.087	0.278
RBC (×10^12^/L)	4.7 ± 1.2	4.9 ± 1.3	1.262	9.208
Hb (g/L)	145.3 ± 13.6	146.9 ± 15.8	0.861	0.390
PLT (×10^9^/L)	213.6 ± 34.7	206.9 ± 35.8	1.495	0.136
NLR	1.91 ± 0.47	1.71 ± 0.55	3.105	0.002
PLR	92.9 ± 22.6	86.2 ± 23.7	2.279	0.024
Cr (µmol/L)	73.8 ± 18.5	70.6 ± 22.1	1.249	0.213
UA (µmol/L)	239.7 ± 44.8	229.6 ± 53.5	1.628	0.105
ALT (U/L)	24.6 ± 5.2	23.5 ± 7.3	1.397	0.164
AST (U/L)	20.3 ± 4.9	21.4 ± 5.5	1.672	0.096
TBIL (µmol/L)	14.4 ± 4.2	13.7 ± 5.1	1.193	0.234
DBIL (µmol/L)	6.6 ± 1.8	6.8 ± 2.3	0.774	0.440
K (mmol/L)	4.67 ± 0.42	4.62 ± 0.48	0.879	0.380
Na (mmol/L)	146.1 ± 9.4	145.5 ± 11.7	0.451	0.652
Cl (mmol/L)	109.3 ± 7.6	107.9 ± 9.2	1.321	0.188
Ca^2+^ (mmol/L)	2.0 ± 0.5	2.1 ± 0.6	1.441	0.151
Hypertension (*n*, %)	12 (8.3)	10 (9.3)	0.089	0.766
Hyperlipidemia (*n*, %)	15 (10.3)	13 (12.1)	0.203	0.652
Diabetes (*n*, %)	7 (4.8)	8 (7.5)	0.772	0.380
Aspirin (*n*, %)	17 (11.7)	9 (8.4)	0.730	0.393

BMI, body mass index; WBC, white blood cell; NEU, neutrophil; LYM, lymphocyte; RBC, red blood cell; Hb, hemoglobin; PLT, platelet; NLR, neutrophil-to-lymphocyte ratio; PLR, platelet-to-lymphocyte ratio; Cr, creatine; UA, uric acid; ALT, alanine aminotransferase; AST, aspartate aminotransferase; TBIL, total bilirubin; DBIL, direct bilirubin.

**Table 3:** Baseline tumor characteristics and treatment between patients with and without tumor progression 红色以下还没改。.

Characteristics	Progression (*n* = 145)	Non-progression (*n* = 107)	*t*/*X*^2^ value	*P*-value
Location			1.304	0.728
Supracerebral lobe (*n* = 185)	107 (73.8)	78 (72.9)		
Supracerebral midline (*n* = 32)	17 (11.7)	15 (14.0)		
Cerebellum (*n* = 27)	15 (10.3)	12 (11.2)		
Brain stem (*n* = 8)	6 (4.1)	2 (1.9)		
Diameter (mm)	29.6 ± 7.1	27.1 ± 8.4	2.555	0.011
Classification			0.152	0.927
Oligodendroglioma	22 (15.2)	18 (16.8)		
Astrocytoma	106 (73.1)	76 (71.0)		
Oligoastrocytoma	17 (11.7)	13 (12.2)		
Operation			17.563	<0.001
Partial resection	83 (57.2)	32 (29.9)		
Total/Enlarged resection	62 (42.8)	73 (70.1)		
Radiotherapy			8.370	0.004
Yes	56 (38.6)	61 (57.0)		
No	89 (61.4)	46 (43.0)		
Chemotherapy			0.001	0.973
Yes	64 (44.1)	47 (43.9)		
No	81 (55.9)	60 (56.1)		

### Risk factors of tumor progression within 5-year follow-up after glioma surgery

Multivariate analysis showed that age >45 years old (OR = 1.35, 95% CI, 1.07–3.19, *P* = 0.027), partial tumor resection (OR = 1.66, 95% CI, 1.15- 3.64, *P* = 0.031), tumor diameter >3 cm (OR = 1.52, 95% CI, 1.14–4.06, *P* = 0.017) and no radiotherapy (OR = 1.37, 95% CI, 1.12–2.44, *P* = 0.039) were independent risk factors of the progression of tumor during the 5-year follow-up period (Table 4).

**Table 4:** Factors associated with tumor progression within 5-year follow-up.

Factors	Univariate analysis			Multivariate analysis		
	OR	95% CI	*P*-value	OR	95% CI	*P*-value
Age >45 years	1.47	1.09–3.38	0.036	1.35	1.07–3.19	0.027
Partial resection	1.72	1.18–2.85	0.025	1.66	1.15–3.64	0.031
Diameter >3 cm	1.63	1.11–3.87	0.033	1.52	1.14–4.06	0.017
No radiotherapy	1.42	1.17–2.56	0.034	1.37	1.12–2.44	0.039

## Discussion

In recent decades, significant progress has been made regarding treatment for glioma, especially for those low-grade gliomas (13–15). But in clinical practice, recurrence is often seen in patients with glioma after tumor resection, which brings big challenge for neurosurgeons (16). The present study retrospectively enrolled patients with low-grade glioma and analyzed the clinical and follow-up data. By univariate and multivariate Logistic regression analysis, we found that there were four predictors for tumor progression within 5 years of follow-up in patients with low-grade glioma after tumor resection: age over 45 years, partial tumor resection, tumor diameter >3 cm, and no radiotherapy after surgery.

Previous findings suggest that patients older than 40 years have a significantly higher risk of recurrence or death after surgery (17, 18). Our results, that age beyond 45 years, were associated with an increased risk of postoperative recurrence of gliomas, were largely consistent with previous studies. The difference is that we analyze the recurrence risk of different ages based on the current age cutoff value for young and middle-aged people, that is, 45 years old, so as to facilitate clinical use. Regarding the predictive value of tumor size for prognosis, our results are that tumors larger than 3 cm in diameter increase the risk of recurrence. Different from the cut-off value of 4 cm or 5 cm in previous studies (19, 20), we believe that the reason may be due to the progress of current diagnostic technology, that more smaller gliomas are found, while the relative ratio of larger gliomas is reduced.

Due to the invasive growth characteristics and the particularity of anatomical location of gliomas, single surgery or radiotherapy cannot avoid tumor recurrence (21, 22). Low-grade gliomas account for 5% of intracranial tumors and can be classified into oligodendrogliomas, astrocytomas, and oligoastrocytomas, and mainly occur in younger patients, especially those in their 20 and 40 s. Imaging examination plays an important role in the diagnosis of glioma (23, 24). Conventional imaging examinations include brain CT and MRI. For the diagnosis of glioma, conventional MRI is superior to CT in images, and MRI can clearly show the extent of invasion of most tumors, surrounding edema, intratumoral hemorrhage, necrosis and Mass effect (25). However, there are still some low-grade gliomas that are not clearly displayed in conventional CT and MRI examinations, which require auxiliary diagnosis such as multimodal imaging, and more importantly, doctors need to carefully identify them during surgery. For low-grade gliomas Surgical treatment is an important treatment method for stromal tumors. For tumors in the non-functional area, the goal of total or subtotal resection is often achieved by means of extended resection (10), while for tumors in the functional area, “maximum safe tumor resection” is used. The strategy is to reduce tumor tissue, reduce tumor compression symptoms, and at the same time protect neurological function as much as possible. The purpose of diagnosis and guidance for further treatment can be achieved by biopsy or partial resection. However, it must be clear that the degree of surgical resection is closely related to the prognosis of patients. A retrospective study including 1097 patients with low-grade glioma showed that, Patients with less than 50% resection had a median survival of 10.5 years; those with 50%–90% resection had a median survival of 14 years, and those with total resection had a median survival of more than 15 years. Therefore, Intraoperative resection of as many tumors as possible under the premise of ensuring safety is beneficial to obtain a better prognosis (26). If the preoperative assessment considers that the degree of tumor resection may be less than 50%, it can be considered biopsy under stereotaxic or navigation (27, 28). Our findings are consistent with those described above. In our study, there was a significant increase in the risk of recurrence during the 5-year follow-up period after partial tumor resection. In recent years, surgery-assistant techniques improved significantly, including neuronavigation, stereotaxic, intraoperative B-ultrasound, intraoperative CT or MRI, etc. to determine the extent of the tumor, as well as intraoperative awakening techniques and brain function localization to protect motor, language, and sensory functions (29, 30). The use of technology has significantly improved the efficacy and safety of surgery.

Due to the aggressive growth pattern of gliomas, surgical treatment alone cannot completely cure diffusely growing tumors (31). At present, adjuvant therapy such as radiotherapy and chemotherapy are used to delay tumor recurrence. Different from high-grade glioma cells, the timing of postoperative radiotherapy and chemotherapy for low-grade glioma is still not standardized, and the need for postoperative radiotherapy and chemotherapy should be assessed according to high-risk factors (32). Currently recognized high-risk factors for low-grade glioma include age greater than or equal to 40 years, tumor diameter greater than or equal to 4 cm, incomplete tumor resection, astrocytoma component, and absence of combined 1p/19q deletion. For those with one of the above high-risk factors, adjuvant therapy such as radiotherapy and chemotherapy is recommended as soon as possible after surgery. However, the regulations on risk factors in various studies are still not unified. In a clinical trial of low-grade glioma in the United States, 111 patients with low-risk factors were observed postoperatively without adjuvant therapy, and the 2-year overall survival rate was 99% and the 5-year overall survival rate was 93%. Therefore, it is still necessary to be cautious about whether radiotherapy and chemotherapy are needed after surgery (33). Due to the location of glioma growth and its aggressive growth pattern, it is difficult to ensure complete resection in every patient with surgical treatment alone. The comprehensive treatment of surgery, radiotherapy and drug therapy is one of the keys to improve the cure rate of glioma (34). Our findings support combined postoperative radiotherapy. In our study, patients who did not undergo radiotherapy also had a significantly higher risk of recurrence during the 5-year follow-up. Surgery and radiotherapy are both local treatments, while drug therapy is systemic therapy. The purpose of further killing tumor cells is achieved through the distribution of drugs throughout the body. The advantage of drug therapy is that it can be performed multiple times. For patients with recurrence who cannot undergo surgery and radiotherapy, drug therapy is often the only treatment method (2). At present, drug therapy includes chemotherapy drug therapy, molecular targeted therapy, biological immunotherapy, etc. However, molecular targeted therapy and biological immunotherapy are still in the clinical trial stage. Eligible and eligible patients should be encouraged to participate in clinical trials of drugs at different disease stages.

Although low-grade gliomas are still difficult to cure completely, compared with other benign intracranial tumors, they still have a higher recurrence rate. However, compared with high-grade glioma, its natural course is longer, and the survival period after comprehensive treatment is significantly longer than that of high-grade glioma patients (35). At present, international treatment methods for low-grade glioma emerge in an endless stream, and various clinical trials continue to be carried out. The clinical research trials under study include postoperative observation, postoperative chemotherapy alone, and clinical efficacy observation of postoperative radiotherapy combined with chemotherapy. Through continuous improvement of surgery and radiotherapy, and the development of new anti-tumor drugs, it is expected to bring better prognosis and longer survival to patients with low-grade gliomas (36).

This study has some limitations: First, this is a single-center, retrospective study with a relatively small sample size, which has a negative influence on the validity of statistical results. Second, we did not include gene examination results or other biomarkers in this study. In our opinion, these biomarkers are not convenient in clinical practice and are expensive for patients. We suggest that future study conduct on multi-center, prospective design to further investigate some factors associated with the prognosis of low-grade glioma.

## Data Availability

The original contributions presented in the study are included in the article/Supplementary Material, further inquiries can be directed to the corresponding author/s.

## References

[1] Chen R Smith-Cohn M Cohen AL Colman H. Glioma subclassifications and their clinical significance. Neurotherapeutics. (2017) 14:284–97. 10.1007/s13311-017-0519-x 28281173 PMC5398991

[2] Louis DN Perry A Wesseling P Brat DJ Cree IA Figarella-Branger D The 2021 WHO classification of tumors of the central nervous system: a summary. Neuro Oncol. (2021) 23:1231–51. 10.1093/neuonc/noab106 34185076 PMC8328013

[3] Ostrom QT Bauchet L Davis FG Deltour I Fisher JL Langer CE The epidemiology of glioma in adults: a “state of the science” review. Neuro Oncol. (2014) 16:896–913. 10.1093/neuonc/nou087 24842956 PMC4057143

[4] Chen W Zheng R Baade PD Zhang S Zeng H Bray F Cancer statistics in China, 2015. CA Cancer J Clin. (2016) 66:115–32. 10.3322/caac.21338 26808342

[5] Pellerino A Bruno F Interno V Ruda R Soffietti R. Current clinical management of elderly patients with glioma. Expert Rev Anticancer Ther. (2020) 20:1037–48. 10.1080/14737140.2020.1828867 32981392

[6] Lin Z Yang R Li K Yi G Li Z Guo J Establishment of age group classification for risk stratification in glioma patients. BMC Neurol. (2020) 20:310. 10.1186/s12883-020-01888-w 32819307 PMC7439690

[7] Weller M Wick W Aldape K Brada M Berger M Pfister SM Glioma. Nat Rev Dis Primers. (2015) 1:15017. 10.1038/nrdp.2015.17 27188790

[8] Ruff M Kizilbash S Buckner J. Further understanding of glioma mechanisms of pathogenesis: implications for therapeutic development. Expert Rev Anticancer Ther. (2020) 20:355–63. 10.1080/14737140.2020.1757440 32301635

[9] Le Rhun E Taillibert S Chamberlain MC. Current management of adult diffuse infiltrative low grade gliomas. Curr Neurol Neurosci Rep. (2016) 16:15. 10.1007/s11910-015-0615-4 26750130

[10] Forst DA Nahed BV Loeffler JS Batchelor TT. Low-grade gliomas. Oncologist. (2014) 19:403–13. 10.1634/theoncologist.2013-0345 24664484 PMC3983820

[11] Jakola AS Unsgard G Myrmel KS Kloster R Torp SH Losvik OK Surgical strategy in grade II astrocytoma: a population-based analysis of survival and morbidity with a strategy of early resection as compared to watchful waiting. Acta Neurochir (Wien. (2013) 155:2227–35. 10.1007/s00701-013-1869-8 24043414

[12] Ganau L Paris M Ligarotti GK Ganau M. Management of gliomas: overview of the latest technological advancements and related behavioral drawbacks. Behav Neurol. (2015) 2015:862634. 10.1155/2015/862634 26346755 PMC4546744

[13] Morshed RA Young JS Hervey-Jumper SL Berger MS. The management of low-grade gliomas in adults. J Neurosurg Sci. (2019) 63:450–7. 10.23736/S0390-5616.19.04701-5 30916536

[14] Semmel D Ware C Kim JY Peters KB. Evidence-based treatment for low-grade glioma. Semin Oncol Nurs. (2018) 34:465–71. 10.1016/j.soncn.2018.10.008 30391119

[15] Hervey-Jumper SL Berger MS. Maximizing safe resection of low- and high-grade glioma. J Neurooncol. (2016) 130:269–82. 10.1007/s11060-016-2110-4 27174197

[16] Nahed BV Redjal N Brat DJ Chi AS Oh K Batchelor TT Management of patients with recurrence of diffuse low grade glioma: a systematic review and evidence-based clinical practice guideline. J Neurooncol. (2015) 125:609–30. 10.1007/s11060-015-1910-2 26530264

[17] Acharya S Lavrador JP Sekhon PP Visagan R Jung J Gullan R Low-grade glioma case series: lessons learned from an evolving 10-year single-institution multidisciplinary team practice. World Neurosurg. (2021) 151:e47–57. 10.1016/j.wneu.2021.03.101 33781942

[18] Fukuya Y Ikuta S Maruyama T Nitta M Saito T Tsuzuki S Tumor recurrence patterns after surgical resection of intracranial low-grade gliomas. J Neurooncol. (2019) 144:519–28. 10.1007/s11060-019-03250-8 31363908

[19] Gui C Lau JC Kosteniuk SE Lee DH Megyesi JF. Radiology reporting of low-grade glioma growth underestimates tumor expansion. Acta Neurochir (Wien). (2019) 161:569–76. 10.1007/s00701-018-03783-3 30756242

[20] Keshri V Deshpande RP Chandrasekhar Y Panigrahi M Rao IS Babu PP. Risk stratification in low grade glioma: a single institutional experience. Neurol India. (2020) 68:803–12. 10.4103/0028-3886.293441 32859817

[21] Ma L Li G Wei M. Neutrophil-to-lymphocyte ratio and its changes are related to grade II–IV glioma recurrence. Cancer Manag Res. (2020) 12:9429–34. 10.2147/CMAR.S267523 33061630 PMC7533248

[22] Teng C Zhu Y Li Y Dai L Pan Z Wanggou S Recurrence- and malignant progression-associated biomarkers in low-grade gliomas and their roles in immunotherapy. Front Immunol. (2022) 13:899710. 10.3389/fimmu.2022.899710 35677036 PMC9168984

[23] Almansory KO Fraioli F. Combined PET/MRI in brain glioma imaging. Br J Hosp Med (Lond). (2019) 80:380–6. 10.12968/hmed.2019.80.7.380 31283392

[24] Maximov II Tonoyan AS Pronin IN. Differentiation of glioma malignancy grade using diffusion MRI. Phys Med. (2017) 40:24–32. 10.1016/j.ejmp.2017.07.002 28712716

[25] Omay SB Piepmeier JM Knisely JP. Low-grade gliomas: when and how to treat. Hematol Oncol Clin North Am. (2012) 26:797–809. 10.1016/j.hoc.2012.05.001 22794284

[26] Mizra FA Shamim MS. Extent of resection and timing of surgery in adult low grade glioma. J Pak Med Assoc. (2017) 67:959–61. 28585606

[27] Mato D Velasquez C Gomez E Marco de Lucas E Martino J. Predicting the extent of resection in low-grade glioma by using intratumoral tractography to detect eloquent fascicles within the tumor. Neurosurgery. (2021) 88:E190–E202. 10.1093/neuros/nyaa463 33313812

[28] Wijnenga MMJ Mattni T French PJ Rutten GJ Leenstra S Kloet F Does early resection of presumed low-grade glioma improve survival? A clinical perspective. J Neurooncol. (2017) 133:137–46. 10.1007/s11060-017-2418-8 28401374 PMC5495869

[29] Chen R Ravindra VM Cohen AL Jensen RL Salzman KL Prescot AP Molecular features assisting in diagnosis, surgery, and treatment decision making in low-grade gliomas. Neurosurg Focus. (2015) 38:E2. 10.3171/2015.1.FOCUS14745 25727224

[30] Mazzucchi E La Rocca G Ius T Sabatino G Della Pepa GM. Multimodality imaging techniques to assist surgery in low-grade gliomas. World Neurosurg. (2020) 133:423–5. 10.1016/j.wneu.2019.10.120 31881559

[31] Bell EH Zhang P Fisher BJ Macdonald DR McElroy JP Lesser GJ Association of MGMT promoter methylation status with survival outcomes in patients with high-risk glioma treated with radiotherapy and temozolomide: an analysis from the NRG oncology/RTOG 0424 trial. JAMA Oncol. (2018) 4:1405–9. 10.1001/jamaoncol.2018.1977 29955793 PMC6117103

[32] Buckner JC Shaw EG Pugh SL Chakravarti A Gilbert MR Barger GR Radiation plus procarbazine, CCNU, and vincristine in low-grade glioma. N Engl J Med. (2016) 374:1344–55. 10.1056/NEJMoa1500925 27050206 PMC5170873

[33] Fisher BJ Hu C Macdonald DR Lesser GJ Coons SW Brachman DG Phase 2 study of temozolomide-based chemoradiation therapy for high-risk low-grade gliomas: preliminary results of Radiation Therapy Oncology Group 0424. Int J Radiat Oncol Biol Phys. (2015) 91:497–504. 10.1016/j.ijrobp.2014.11.012 25680596 PMC4329190

[34] Liang S Fan X Zhao M Shan X Li W Ding P Clinical practice guidelines for the diagnosis and treatment of adult diffuse glioma-related epilepsy. Cancer Med. (2019) 8:4527–35. 10.1002/cam4.2362 31240876 PMC6712518

[35] Sepulveda-Sanchez JM Munoz Langa J Arraez MA Fuster J Hernandez Lain A Reynes G SEOM Clinical guideline of diagnosis and management of low-grade glioma (2017). Clin Transl Oncol. (2018) 20:3–15. 10.1007/s12094-017-1790-3 29124520 PMC5785601

[36] van den Bent MJ Afra D de Witte O Ben Hassel M Schraub S Hoang-Xuan K Long-term efficacy of early versus delayed radiotherapy for low-grade astrocytoma and oligodendroglioma in adults: the EORTC 22845 randomised trial. Lancet. (2005) 366:985–90. 10.1016/S0140-6736(05)67070-5 16168780

